# Next-Generation Sequencing-Based Detection of *KRAS* G12D Variants in Colorectal Cancer: A Retrospective Cohort Study

**DOI:** 10.3390/genes17020174

**Published:** 2026-01-31

**Authors:** Gulam Hekimoglu, Metin Eser, Murat Hakki Yarar, Fatma Gulcicek Ayranci, Melike Ozcelik

**Affiliations:** 1 Department of Histology and Embryology, International Faculty of Medicine, University of Health Sciences, Istanbul 34668, Turkey; 2 Department of Medical Genetics, Umraniye Education and Research Hospital, University of Health Sciences, Istanbul 34766, Turkey; esermetin325@gmail.com (M.E.); hakkiyarar8@gmail.com (M.H.Y.); 3 Department of Medical Pathology, Umraniye Education and Research Hospital, University of Health Sciences, Istanbul 34766, Turkey; gulcicekayranci@gmail.com; 4 Department of Medical Oncology, Umraniye Education and Research Hospital, University of Health Sciences, Istanbul 34764, Turkey

**Keywords:** *KRAS* G12D, *BRAF*, NGS, CNV, colorectal cancer

## Abstract

**Purpose**: Colorectal cancer (CRC) is a highly aggressive malignancy of the digestive system. Somatic variants in the Kirsten rat sarcoma virus oncogene homolog (*KRAS*) gene have a significant influence on CRC progression and serve as key predictors of resistance to anti-epidermal growth factor receptor (EGFR) therapy. This study aimed to determine the prevalence of *KRAS* variants, with a particular focus on G12D variants, which represent potential for targeted therapy. **Methods**: A cohort of 73 CRC patients was evaluated between January 2021 and August 2024. Next-generation sequencing (NGS) was performed using the Archer^®^ VariantPlex^®^ Solid Tumor Focus v2 (Integrated DNA Technologies, Inc., Boulder, CO, USA) assay on the Illumina NextSeq platform. The gene panel included 20 genes frequently mutated in solid tumors, assessing point variants, insertions/deletions, and microsatellite instability. **Results**: The cohort of the study comprised 38 female (52%) and 35 males (48%) patients aged 31–83 years (mean, 58.77 ± 12.72). No significant difference in mean age was observed between males and females (60.31 ± 12.32 vs. 57.34 ± 13.08; *p* > 0.05). *KRAS* variants were detected in 30 patients (41%). Among these, the variant frequencies for G12D, G12V, and G13D were 7%, 11%, and 11%, respectively. Additionally, one patient (1.4%) harbored an *ERBB2* amplification. All *KRAS* variants were associated with resistance to anti-*EGFR* therapy. Notably, *KRAS* G12D variants have potential responsiveness to targeted therapy, while human epidermal growth factor receptor 2 (*ERBB2*) amplifications are responsive to anti-HER2 treatments and resistant to anti-*EGFR* therapies. **Conclusions**: These findings highlight the clinical significance of *KRAS* variant profiling for prognosis and personalized treatment planning in CRC. Moreover, assessing *KRAS* variants individually is crucial to better understanding treatment response and exploring the potential targeted therapy in CRC management.

## 1. Introduction

Colorectal cancer (CRC) is a highly aggressive cancer type of the digestive system. Patients who receive a diagnosis at an advanced stage are more likely to have a bad prognosis [1]. Monoclonal antibodies that block the signaling cascade started by the binding of the epidermal growth factor to its receptor (EGFR) are used in certain targeted treatments for CRC. Somatic variants in genes like the rat sarcoma (*RAS*) and the v-Raf murine sarcoma viral oncogene homolog B1 (*BRAF*) that are involved in the EGFR signaling cascade dictate how well this therapy works. In the absence of RAS variants, EGFR monoclonal antibody (anti-EGFR) therapy combined with chemotherapy may be beneficial for the patients as a first-line treatment. Numerous chromosomal abnormalities linked to CRC have been identified thanks to new genomic approaches. As a growth signal transducer downstream of the EGFR, Kirsten rat sarcoma virus oncogene homolog (*KRAS*), an oncogene that is a member of the GTPase *RAS* superfamily, initiates processes related to cell division and proliferation. Therefore, KRAS plays a role in neoplastic transformation as an early event in colorectal carcinogenesis when it is overactive due to a genetic alteration. Research has indicated that the frequency of variants in RAS in patients with CRC differs by community [2,3,4]. While Neuroblastoma *RAS* viral oncogene homolog (*NRAS*) variants range from 1% to 7% in frequency, the range of *KRAS* gene variants is 27% to 56% [5]. RAS variant status, clinicopathological characteristics, and demographic information have all been linked in a number of investigations. Variants in the *KRAS* gene, for example, have been connected to the patient’s age, sex, and tumor histology [6,7]. Metastasis, decreased survival, and poor prognosis are all linked to variants in *KRAS* [8,9]. Another member of the RAS family, the v-raf murine sarcoma b-viral oncogene (*BRAF*), is an essential protein kinase in the mitogen-activated protein kinase MAPK/ERK signaling pathway. Proliferation, angiogenesis, migration, and cell differentiation are all linked to BRAF activation. Products of the *BRAF* gene function in the MAPK signaling pathway downstream of *KRAS*. The MEK–ERK pathway is abnormally activated when an amino acid in codon 600 (V600E) is substituted from valine to glutamic acid. The most significant change is *BRAF* V600E, which accounts for 90% of all *BRAF* variants and is a sign of severe disease [10]. Additionally, *BRAF* variants are linked to poor prognosis and decreased survival rates, particularly in cancers that have microsatellite instability [11,12]. Despite being a poor prognostic factor for metastatic cancer [13], BRAF V600E is a promising target for personalized treatment, and CRC is more effectively treated when certain BRAF V600E inhibitors are combined with other MAPK/PI3K pathway inhibitors [14].

As a biomarker for advanced cancer patients to assess their suitability for immune checkpoint inhibitors (ICIs), microsatellite instability (MSI) is attracting more and more attention. Short DNA sequences of repeating nucleotides with a high mistake rate are known as microsatellites. One method of testing would be to use a molecular test, like multiplex PCR, to screen the CRC and verify the MSI status [15]. MSI is caused by inadequate DNA mismatch repair. Clinical trials have examined the utility of immunotherapy and preoperative treatment for patients with MSI-high CRC [10]. The National Cancer Institute recommends using a panel of five microsatellite loci to evaluate MSI status [16]. A high MSI status is associated with a *KRAS* or *BRAF* variant [17]. Variants in *KRAS* indicate that anti-EGFR monoclonal antibodies (mAb) cetuximab and panitumumab have low therapeutic efficacy because of resistance, suggesting a diverse dependency of *KRAS*-variant cancers on specific *KRAS* variant alleles [18].

*Tumor protein 53* (*TP53*) is one of the most frequently altered genes in CRC, playing a crucial role in DNA synthesis and repair, genome stability, apoptosis or senescence, and growth arrest [19]. Alongside *KRAS* and *TP53*, amplification in the *ERBB2* proto-oncogene—though rare—is also observed in CRC. The human epidermal growth factor receptor 2 (HER2), encoded by *ERBB2*, is a membrane-bound receptor whose gene amplification has been identified in certain CRC xenograft models resistant to cetuximab, even in the absence of *KRAS*, *NRAS*, or *BRAF* variants [20]. Using NGS, MSI, and copy number variation (CNV) testing, we aimed to evaluate the frequency of *KRAS* variants in CRC and their association with anti-EGFR therapy response. Given that *KRAS* variants contribute to drug resistance and poor prognosis, determining variant status and associated clinicopathological features is vital for optimizing CRC management and patient follow-up. Accordingly, this study sought to determine the prevalence of *KRAS* variants and explore their potential relationship with targeted inhibitor therapies.

## 2. Materials and Methods

### 2.1. Patients

A total of 73 CRC patients were examined. From January 2021 to August 2024, the patients were chosen from the Umraniye Education and Research Hospital. The following were the study’s inclusion criteria: (1) age over 18 years, (2) cases with confirmed pathogenic colorectal adenocarcinoma, (3) individuals without tumors in a different focus than colorectal adenocarcinoma. Written informed consent for genetic analysis and processing of patient data was obtained from all participants. The Ethics Committee of Umraniye Education and Research Hospital gave its approval to the project. The patients’ clinically stage III, IV colorectal adenocarcinoma biopsy or surgical materials were evaluated.

### 2.2. Next-Generation Sequencing

Initially, the primary sample’s DNA was isolated to amplify genes, gene sections, or fusions linked to the condition. The extracted DNA sample underwent quality control. The DNA-based “The Archer^®^ VariantPlex^®^ Solid Tumor Focus v2” kit was used to amplify the target areas, and the Illumina NextSeq platform (Illumina, San Diego, CA, USA) was used to sequence them using the NGS technique. Archer Analysis (Version: 7.5.1, Copyright 2014–2026, Integrated DNA Technologies, Inc.) software was used to conduct secondary analyses (data cleaning, alignment, and variant/fusion identification) on the acquired raw data (Fastq). The alignment was performed using the human reference genome, hg19 (GRCh37). Deduplicated reads are used for accurate variant/fusion and quantitative multiplex data analysis, whereas molecular barcodes are used for false correlations in the Archer Analysis program. The goal of the next-generation sequencing NGS test, the Archer^®^ VariantPlex^®^ Solid Tumor Focus v2 kit, is to find and identify point variants, indels, and microsatellite instability in 20 genes that are commonly mutated in solid tumors. They employed a solid tumor focus panel and analyzed it. *AKT1*, *BRAF*, *EGFR*, *ERBB2*, *FOXL2*, *GNA11*, *GNAQ*, *GNAS*, *HRAS*, *IDH1*, *IDH2*, *KIT*, *KRAS*, *MET*, *NRAS*, *PDGFRA*, *PIK3CA*, *RET*, *TERT*, and *TP53* were among those genes. Even without prior knowledge of known fusion partners or breakpoints, the kit detects fusions of all target genes in a single sequencing test using Archer’s exclusive Anchored Multiplex PCR (AMPTM)-based enrichment. For both known and undiscovered variants, the technique makes use of unidirectional, gene-specific primers (GSPs). Both patient indexes and molecular barcodes are present in the barcodes. The “HeatMap” graphic provides expression levels.

### 2.3. Microsatellite Instability

For each of the 114 microsatellite loci, the MSI algorithm determines the Shannon diversity (instability score) of the repeat lengths in distinct reads. The instability score of each locus is compared to the instability scores of a baseline cohort of stable samples included in the pipeline to assess the locus’s stability. At each locus in the interrogated sample, the baseline samples are scaled to correspond with the sequencing depth. Depending on the user’s preferences, the percentage of loci found to be unstable influences whether samples are classified as microsatellite instability-high (MSI-H), microsatellite instability intermediate, or microsatellite stable (MSS). Based on the total percentage of unstable microsatellite (MS) loci, the MSI pipeline determines the microsatellite stability for each sample: MS-Stable: less than 20% of loci are unstable, MS-Intermediate: 20–30%, and MS-High: greater than 30%. To make sure every locus satisfies a minimum read depth threshold of 25, a read depth quality control check is performed per-MS locus. To make sure every sample has at least 35,000 total acceptable MS readings, a second quality control check is performed. Samples will be classified as MSI-Unknown if they do not pass either of these quality control tests.

### 2.4. Copy Number Variation

The CNV assay compares the copy number of normal and disease samples using one or more control samples, if any. Every sample in the task is utilized as a baseline if there are no controls. The analysis software uses this data to generate a copy number readout and visualization, as well as a *p*-value to indicate call confidence. Every primer with CNV enabled in a panel has its copy number determined; these primers are represented by dots in the CNV grid. The outcomes of these primer-by-primer analyses are then compiled and shown for more extensive genomic areas.

### 2.5. Data Analysis

First, the data quality of the raw data that was moved to the Archer Analysis platform was assessed. Following a review of these measures, appropriate samples were added to the analytical flow and assessed in relation to the designated disease. Taking into account the patient’s clinical data, filtering was performed in the analysis flow to scan alterations associated with the designated tumor kind. Following filtering, changes to the lists were categorized based on whether they were found in databases and guidelines and contained information about diagnosis, prognosis, and therapy. The Association for Molecular Pathology/American Society of Clinical Oncology/College of American Pathologists (AMP/ASCO/CAP) recommendations [21] and the evidence about the alterations were taken into consideration while classifying.

### 2.6. Classification and Evidence Levels

Tier I: (A) Variants in a particular tumor type that are both sensitive to and resistant to Food and Drug Administration (FDA)-approved and guideline-approved therapies. (B) Variants included in extensive, well-researched investigations by professional organizations. Tier II: (C) Variants in a different tumor type that are both sensitive to and resistant to FDA-approved and guideline-approved therapies. (D) Variants are featured in preclinical research or case reports. Tier III: Variants with unclear clinical implications. Tier IV: Variants with extremely high allele frequencies have not been linked to a particular malignancy [21].

### 2.7. NGS Quality Metrics

The quality metrics used in this NGS study were selected to ensure high analytical accuracy, reliability of variant detection, and clinical relevance of the reported findings. Each parameter is described in detail below: A minimum sequencing depth threshold was applied, and only samples achieving at least 2.5 million total reads per sample were included in the analysis. This criterion ensured adequate coverage across all targeted regions and reduced the risk of false-negative variant detection. Alternate Observations (AO) represent the total number of sequencing reads supporting the presence of an alternate allele. A minimum AO value of 5 was required to minimize the possibility that detected variants resulted from random sequencing errors or background noise. Unique Alternate Observations (UAO) indicate the number of independent sequencing reads derived from unique molecular fragments that support the alternate allele. A threshold of at least 3 unique observations was applied to increase confidence in true variant calls and to reduce the influence of PCR duplication artifacts. Population allele frequency filtering was performed using the Genome Aggregation Database (gnomAD). Variants with a gnomAD allele frequency of 0.05 or higher were excluded, as such variants are considered common polymorphisms and are unlikely to be clinically significant in the context of this study. Variant Allele Fraction (AF) reflects the proportion of sequencing reads supporting the alternate allele relative to the total number of reads at a given genomic position. An AF threshold of at least 0.027 was applied to ensure that detected variants were present at a biologically meaningful level, particularly in samples with low tumor cellularity or circulating tumor DNA. Finally, an allele fraction outlier statistical test was applied. Variants with an AF outlier *p*-value below 0.01 were considered statistically significant, indicating that the observed allele fraction was unlikely to be due to random variation. This criterion provided an additional level of statistical confidence in variant detection. Together, these quality control parameters enhanced the robustness of the NGS analysis, optimized variant calling accuracy, and ensured the reliability of downstream clinical and research interpretations.

### 2.8. Statistical Analysis

GraphPad Prism 8.4.2 was used to analyze the data. Descriptive statistics were used to summarize baseline demographic and clinicopathological characteristics. Categorical variables were compared using Fisher’s exact test or the chi-squared test, as appropriate. All statistical tests were two-tailed, and a *p*-value < 0.05 was considered statistically significant.

## 3. Results

### 3.1. Clinical Characteristics of Patients’ Cohort and Frequencies of RAS Variants

This is a retrospective study of patients with CRC. At the time of testing, the 73 CRC patients ranged in age from 31 to 83 years, with a mean age of 58.77 ± 12.72. There were 38 (52%) female and 35 (48%) male patients. Men and women had nearly identical mean ages (60.31 ± 12.32 vs. 57.34 ± 13.08). Of the patients, 30 out of 73 (41%) tested positive for *KRAS* variants. We compared the *KRAS* variant status between different genders and between those over and under 65 years of age. No significant differences were found between them. The results are shown in Table 1.

**Figure 1:**
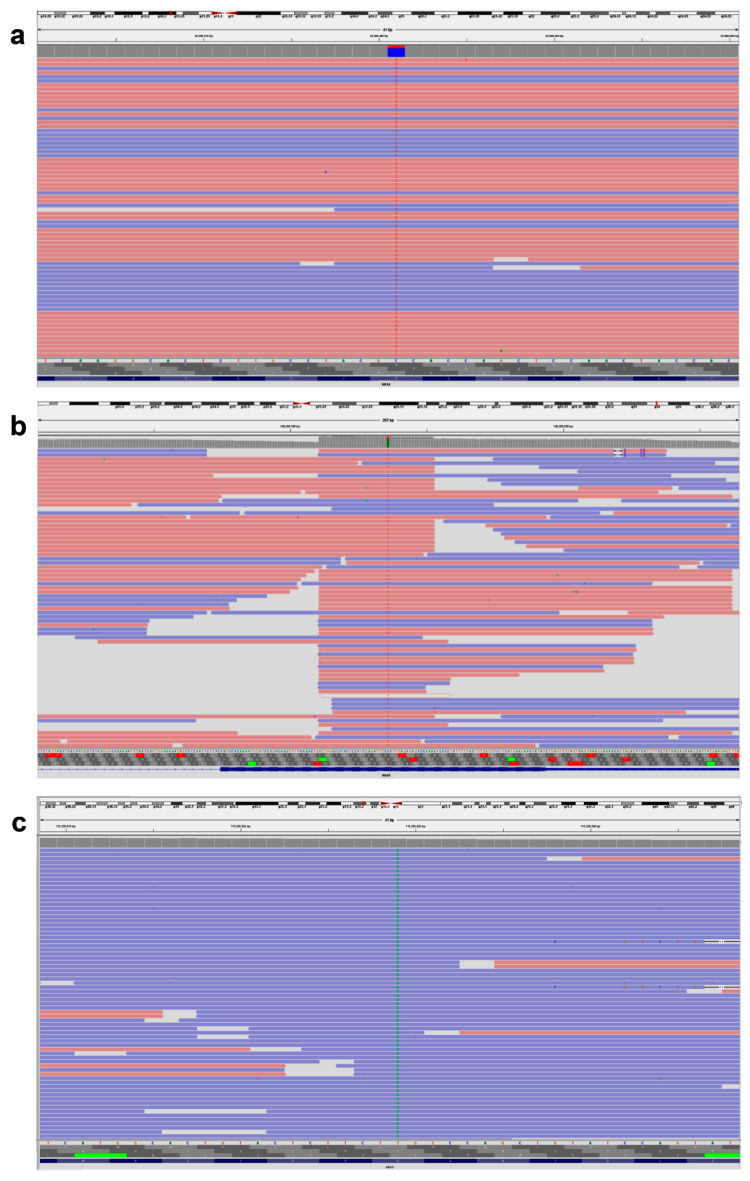
Representative IVG image of *KRAS*, *BRAF*, and *NRAS* gene variants. (**a**) *KRAS*, NM_004985.5 c.38G > A (p.Gly13Asp). (**b**) *BRAF*, NM_004333.4 c.1799T > A (p.Val600Glu). (**c**) *NRAS*, NM_002524.4 c.182A > T (p.Gln61Leu). The pink background indicates sequences read forward, and the light blue indicates sequences read reverse.

Our investigation found that exon 2 included 21 (70%) *KRAS* variants, exon 3 contained 3 (10%) *KRAS* variants, and exon 4 contained 6 (20%) *KRAS* variants. The *KRAS* variants in codon 12 were more common than those in codon 13 (43% vs. 27%, Table 2). For our group, c.35G > T p.Gly12Val (G12V 8/30, 26.6%) was the most frequently reported variant in exon 2, followed by c.35G > A p.Gly12Asp (G12D 5/30, 16.6%). According to Table 2, every variant in codon 13 of exon 2 was of the form c.38G > A p.Gly13Asp (G13D 8/30, 26.6%) (Figure 1a).

The *KRAS* exon 3 variants at codon 61 were detected in only two patients (Table 2). c.183A > C p.Gln61His (Q61H) and c.183A > T p.Gln61His (Q61H) were variants in 61 codons. No individuals tested positive for *KRAS* variants in exon 3’s codon 59. Every patient who tested positive for *KRAS* exon 4 had codon 146 variants, specifically c.436G > A p.Ala146Thr (A146T). *KRAS* variants in codon 117 of exon 4 were not detected in any of the subjects (Table 3). Every *BRAF* variant found was a variation of c.1799T > A p.Val600Glu (V600E) (Figure 1b). Four *NRAS* variants were discovered overall, and one patient had co-variants in both *NRAS* and *KRAS*. One sample had *NRAS* variants in exon 2 (codon 13), three samples had exon 3 (codon 61), and none of the samples had exon 4 (codon 146 or 117) (Figure 1c). Analysis of treatment response showed that all 30 *KRAS* variants and 4 *NRAS* variants were resistant to the FDA-approved medications cetuximab, panitumumab, and tutatinib + trastuzumab. In contrast, cetuximab + encorafenib therapy showed a positive response in patients with *BRAF* variants.

**Figure 2:**
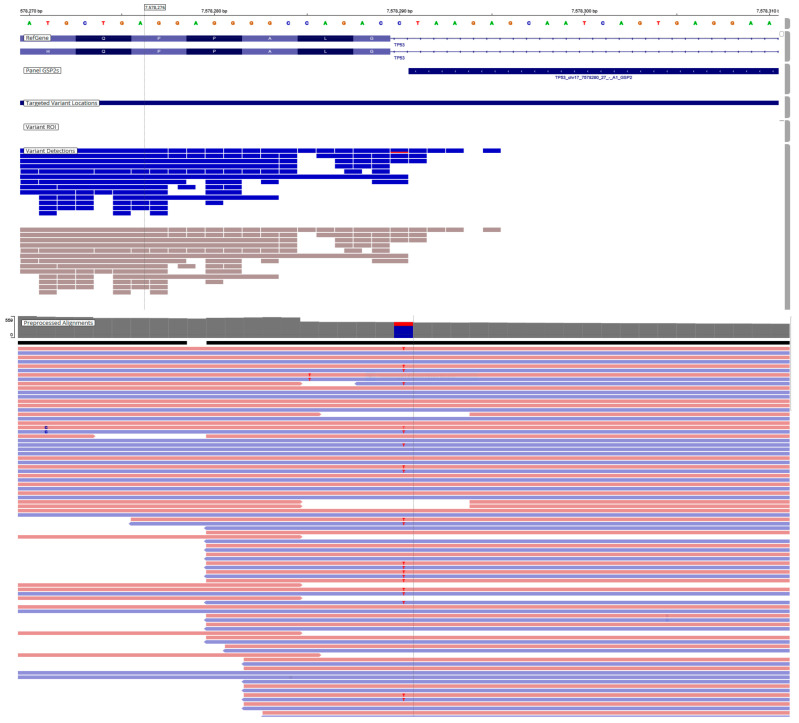
Representative IVG image of *TP53* gene variants. *TP53*, NM_000546.6, c.560-1G > A p.(?). Classification according to the AMP/ASCO/CAP guideline, Tier-IB. The pink background indicates sequences read forward, and the light blue indicates sequences read reverse.

Additionally, 45.2% (33/73) of *TP53* variants (Figure 2) were found in our cohort, either alone or in association with other genes such as *KRAS*, *BRAF*, *PIK3CA*, and *NRAS*. *ERBB2* amplification was identified in 1.4% (1/73) of the cases (Table 3).

### 3.2. Microsatellite Instability Evaluation

**Figure 3:**
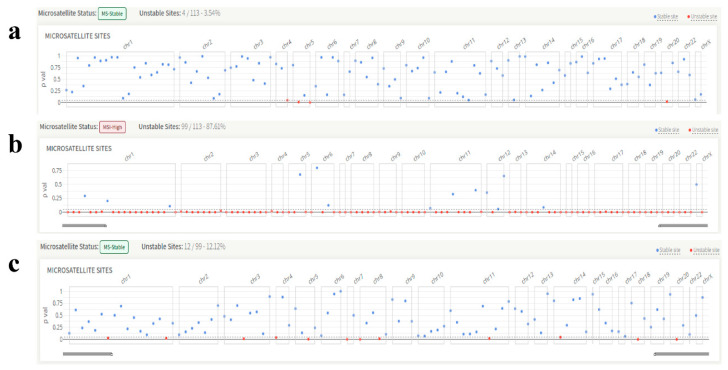
Representative images of the microsatellite instability status of the patients. (**a**) *KRAS*, exon 2, codon 13 variant, microsatellite instability: stable. (**b**) *BRAF V600E* variant, microsatellite instability: unstable. (**c**) *NRAS*, exon 3, codon 61 variant, microsatellite instability: stable.

All but two patients had a stable MSI status according to MSI testing. Representative *KRAS* (Figure 3a), *BRAF* (Figure 3b), and *NRAS* (Figure 3c) genes’ microsatellite instability images are shown below. In addition, Tier IA variations were found in 35 out of 73 patients classified using the AMP/ASCO/CAP standards.

### 3.3. Copy Number Variation Assessment

**Figure 4:**
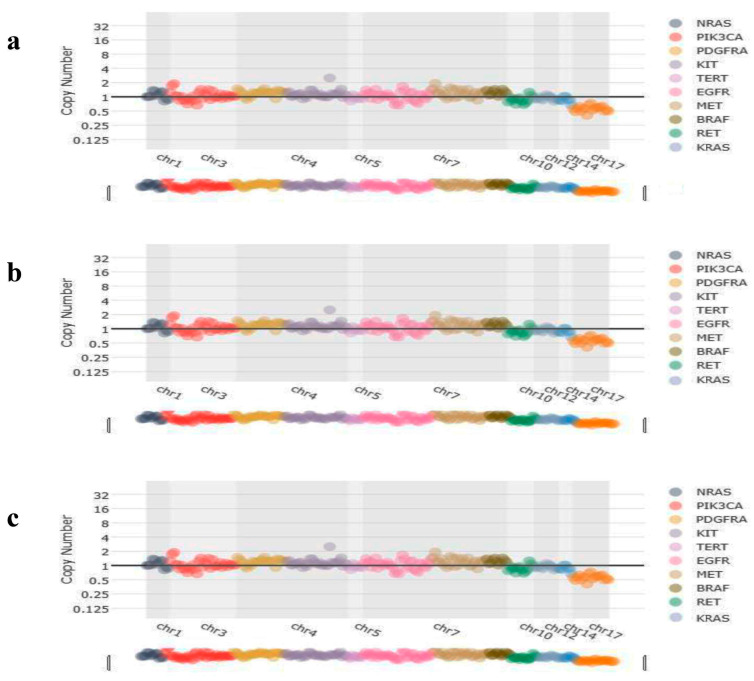
Representative image of copy number variations of the patients. (**a**) No copy number variations were detected in the CNV analysis of *KRAS*-mutated patients. (**b**) No copy number variations were detected in the CNV analysis of *BRAF*-mutated patients. (**c**) No copy number variations were detected in the CNV analysis of *NRAS*-mutated patients.

**Figure 5:**
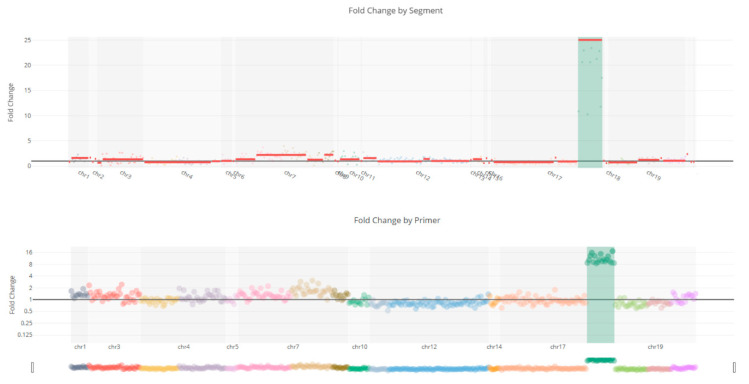
Copy number variation of the *ERBB2* gene shows a 25-fold amplification in the *ERBB2* gene.

CNV analysis results showed that no copy number variations were detected in KRAS (Figure 4a), BRAF (Figure 4b), and NRAS (Figure 4c) patients, but found amplification of the ERBB2 (Figure 5) in a case.

## 4. Discussion

In this investigation, we found that 41% of our CRC patients had *KRAS* variants, consistent with a recent study [22]. Among these *KRAS* variants, G12V was the most frequently reported variant at codon 12 of exon 2, followed by G12D. Every variant in exon 2’s codon 13 was of the G13D. As G12D and G12V compete for the top rank, recent studies demonstrated that the *KRAS* G12D variant is the most prevalent, accounting for 10–12% of CRC cases [23]. Studies have shown that *EGFR* inhibitors and *KRAS* G12D blockers have synthetic lethal effects because simultaneous objecting of *KRAS* G12D and *EGFR* can significantly enhance in vitro apoptosis and in vivo tumor shrinkage. The investigation of the combined *KRAS* G12D/*EGFR* restraint method to enhance responsiveness to therapy and increase benefits for patients with *KRAS* G12D-variant CRC. *KRAS* mutation activation is common in CRC, lung cancer other than small cell carcinoma, and pancreatic cancer. Genetic research has shown that certain variant *KRAS* alleles are linked to distinct tissue-specific genetic dependencies in addition to unique signaling characteristics associated with each mutant form of *KRAS* [24], underscoring the complexity underlying the disparate clinical outcomes of *KRAS* allele-specific inhibitors. For instance, sotorasib, the first approved *KRAS* G12C variation-selective inhibitor, produced a long-lasting therapeutic benefit for patients with *KRAS* G12C-variant lung cancer, excluding small cell carcinoma, while those with CRC carrying the same variant responded less well to the same medication [25]. An emerging therapeutic approach to overcome adaptive resistance in patients with these particular *KRAS* variants is the combination of *EGFR* inhibitors with *KRAS* G12C or G12D inhibitors. Due to compensatory overexpression of *EGFR* signaling pathways, research has demonstrated that *KRAS* G12C inhibitors, such as sotorasib or adagrasib, alone frequently have limited efficacy in CRC [18]. Thus, better response rates and disease control have been seen in preclinical models and early-phase clinical trials when *KRAS* and *EGFR* are blocked simultaneously [26].

We found that 45.2% (33/73) of our cohort carried *TP53* variants, either alone or in combination with other oncogenic genes such as *NRAS*, *BRAF*, phosphatidylinositol-4,5-bisphosphate 3-kinase catalytic subunit alpha (*PIK3CA*), and *KRAS*. Recent studies indicate that several experimental compounds are being developed to correct *TP53* variants or to restore the functional activity of the p53 protein. These include approaches utilizing small molecules that reactivate mutant p53 and agents targeting the MDM2–p53 interaction [27]. One such compound, Eprenetapopt (APR-246), has been investigated in cancer harboring *TP53* variants but has not yet received FDA approval as a targeted therapy [28]. Furthermore, the investigational compound JAB-30300 has received FDA IND approval for a Phase 1/2a clinical trial targeting advanced solid tumors harboring the *p53* Y220C variant [29]. Studies have demonstrated that the NGS technology employed for detecting *ERBB2* gene amplification offers significantly greater sensitivity and precision compared to traditional immunohistochemistry (IHC) and fluorescence in situ hybridization (FISH) methods. In our study, *ERBB2* amplification was detected in 1.4% (1/73) of cases, aligning with previous findings that reported a similar frequency of around 2% [30]. *ERBB2* CNV is recognized as a predictive biomarker for treatment response and can be identified in both tumor tissue and plasma samples. In 2024, the FDA granted tumor-agnostic accelerated approval to trastuzumab deruxtecan (Enhertu) for the treatment of HER2-positive solid tumors [20], marking a significant advancement by allowing its use across multiple cancer types with *ERBB2*/*HER2* amplification [31].

In our study, biopsy or surgical specimens were obtained from patients with a confirmed pathological diagnosis and clinically staged stage III–IV disease, ensuring high diagnostic accuracy and strengthening the reliability of our findings. Building on this well-defined pathological framework, recent advances in deep learning (DL) have demonstrated substantial potential to further enhance the analysis of histopathology images for CRC diagnosis and classification. An increasing amount of research shows that DL-based algorithms, especially transformer-based models and convolutional neural networks (CNNs), can reliably differentiate between benign and malignant tissues, frequently attaining performance metrics that are on par with or better than those of seasoned pathologists in sizable multicenter datasets. AI models trained on whole-slide histopathology images, for instance, have demonstrated strong agreement with expert diagnoses and excellent diagnostic accuracy, indicating useful support tools for standard clinical pathology operations [32]. The broad applicability of DL approaches in CRC histopathology is further demonstrated by systematic reviews and scoping analyses, which show that they not only help with binary cancer classification but also enable more granular tumor categorization and the extraction of clinically relevant features from CRC histology images [33]. Ongoing research indicates that deep learning models could be incorporated into CRC diagnostic workflows to increase accuracy, efficiency, and reproducibility with additional refinement and rigorous clinical validation, even though there are still issues with external validation and generalizability across various clinical settings [34].

In this regard, by improving risk stratification and diagnostic precision, the incorporation of deep learning-based algorithms into screening data and histopathological analysis may further improve early CRC identification [35]. A more individualized, efficient, and prognostically informative approach to CRC prevention and therapy can be achieved by combining the use of artificial intelligence, genetic counseling, and molecular testing with traditional screening methods. In addition, healthcare professionals are our target group for CRC prevention because they can lower their personal risk of developing the disease and increase their ability to advocate for evidence-based screening procedures. Genetic testing and genetic counseling are essential for identifying people with hereditary CRC syndromes, such as Lynch syndrome, and for directing treatment choices, surveillance levels, and preventive measures, in addition to stool-based tests and colonoscopies that are advised by guidelines. Personalized treatment choices are made possible by early detection of harmful mutations, which may also enhance prognosis and clinical results [36].

As this was a retrospective study conducted at a single genetics center, there were limitations in patient follow-up and access to comprehensive clinical data. In addition, the sample size was relatively limited. Future multicenter studies involving larger patient cohorts are planned to address these limitations and to strengthen the generalizability of the findings.

In conclusion, our study confirms that *KRAS* variants, especially G12C and G12D, are highly prevalent in CRC and are associated with poor prognosis and limited therapies. Combining *EGFR* inhibitors with *KRAS* G12C/G12D-targeted agents may help overcome resistance. Additionally, pathogenic *TP53* variants and *ERBB2* amplification were observed, with available FDA-approved or investigational therapies, offering guidance for treatment decisions and genetic counseling.

## Figures and Tables

**Table 1:** Relationship between *KRAS* variant status and clinical characteristics.

Clinical Characteristics		Numbers (%)	*KRAS* Variant Status		*p*-Value
			Wild-Type n (%)	Variant n (%)	
Sex	Female	38 (52%)	21 (48.84)	17 (56.66)	
	Male	35 (48%)	22 (51.16)	13 (43.33)	0.6349
Age	<65	47 (64.38%)	31 (72.09)	16 (53.33)	
	≥65	26 (35.62%)	12 (27.91)	14 (46.66)	0.1369

**Table 2:** Types of variants of the *KRAS* gene detected in CRC patients.

Exons	Codons	Type of Variants	Number of *KRAS* Variants (%)
2	12	c.35G > A (G12D)	5 (16.66)
2	12	c.35G > T (G12V)	8 (26.66)
2	13	c.38G > A (G13D)	8 (26.66)
3	61	c.183A > C (Q61H)	2 (6.66)
3	61	c.183A > T (Q61H)	1 (3.33)
4	146	c.436G > A (A146T)	6 (20.00)

**Table 3:** Genetic and drug sensitivity information of CRC patients.

Case No.	Gender /Age	Gene	Transcript	Variant /Protein	MSI	COSMIC	Classification According to AMP/ASCO/CAP Guideline	Response /Resistance
**1**	M/67	No	No	No	Stable	No	No	Yes
**2**	M/66	KRAS	NM_004985.4	c.35G > T p.Gly12Val	Stable	COSM520	TierIA	/Yes
**3**	F/59	No	No	No	Stable	No	No	No/No
**4**	M/69	KRAS	NM_004985.4	c.38G > A p.Gly13Asp	Stable	COSM532	TierIA	/Yes
**5**	F/71	KRAS	NM_004985.4	c.436G > A p.Ala146Thr	Stable	COSM19404	TierIA	/Yes
**6**	M/61	PIK3CA	NM_006218.3	c.3140A > G p.His1047Arg	Stable	COSM775	TierIIC	No/No
**7**	F/39	KRAS TP53	NM_004985.4 NM_000546.5	c.35G > T p.Gly12Val c.532C > G p.His178Asp	Stable	COSM520 COSM44901	TierIA	/Yes
**8**	F/35	No	No	No	Stable	No	No	No/No
**9**	M/62	KRAS	NM_004985.4	c.436G > A p.Ala146Thr	Stable	COSM19404	TierIA	/Yes
**10**	M/42	NRAS TP53	NM_002524.4 NM_000546.5	c.182A > T p.Gln61Leu c.524G > A p.Arg175His	Stable	COSM583 COSM10648	TierIA TierIII	/Yes
**11**	F/55	PIK3CA	NM_006218.3	c.33140A > G p.His1047Arg	Stable	COSM775	TierIIC	No/No
**12**	F/50	KRAS PIK3CA	NM_004985.4 NM_006218.3	c.38G > A p.Gly13Asp c.3140A > G p.His1047Arg	Stable	COSM532 COSM775	TierIA TierII	/Yes
**13**	F/44	KRAS	NM_004985.4	c.35G > A p.Gly12Asp	Stable	COSM521	TierIA	/Yes
**14**	M/73	TP53	NM_000546.5	c.524G > A p.Arg175His	Stable	COSM10648	TierIII	No/No
**15**	F/45	KRAS TP53	NM_004985.4 NM_000546.5	c.35G > T p.Gly12Val c.743G > A p.Arg248Gln	Stable	COSM520 COSM99602	Tier IA TierIII	/Yes
**16**	F/63	KRAS	NM_004985.4	c.35G > A p.Gly12Asp	Stable	COSM521	TierIA	/Yes
**17**	M/50	PIK3CA TP53	NM_006218.3 NM_000546.5	c.1258T > C p.Cys420Arg c.266_270del p.Pro89LeufsTer58	Stable	COSM757 No	TierIIC TierIII	No/No
**18**	M/51	KRAS PIK3CA TP53	NM_004985.4 NM_006218.3 NM_000546.5	c.436G > A p.Pro89LeufsTer58 c.1258T > C p.Cys420Arg c.742C > T p.Arg248Trp	Stable	COSM19404 COSM757 COSM10656	TierIA TierIIC TierIII	/Yes
**19**	M/33	No	No	No	Stable	No	No	No/No
**20**	F/77	KRAS PIK3CA	NM_004985.4 NM_006218.3	c.35G > T p.Gly12Val c.1633G > C p.Glu545Gln	Stable	COSM520 COSM27133	TierIA TierIIC	/Yes
**21**	F/67	KRAS	NM_004985.4	c.35G > T p.Gly12Val	Stable	COSM520	TierIA	/Yes
**22**	F/59	TP53	NM_000546.5	c.726C > A p.Cys242Ter	Stable	COSM44378	TierIII	
**23**	F/62	KRAS	NM_004985.5	c.38G > A p.Gly13Asp	Stable	COSM532	TierIA	/Yes
**24**	F/31	TP53	NM_000546.6	c.844C > T p.Arg282Trp	Stable	COSM3378339	Tier-III	No/No
**25**	M/74	NRAS TP53	NM_002524.5 NM_000546.6	c.181C > A p.Gln61Lys c.583A > T p.Ile195Phe	Stable	COSM580 COSM44633	Tier-IA Tier-IB	/Yes
**26**	M/59	No	No	No	Stable	No	No	No/No
**27**	F/43	KRAS TP53	NM_004985.5 NM_000546.6	c.436G > A p.Ala146Thr c.517G > C p.Val173Leu	Stable	COSM1904 COSM44057	TierIA TierIB	/Yes
**28**	F/70	No	No	No	Stable	No	No	No/No
**29**	M/67	BRAF	NM_004333.4	c.1799T > A p.Val600Glu	Stable	COSM476	Tier-IA	Yes/
**30**	M/46	TP53	NM_000546.6	c.844C > T p.Arg282Trp	Stable	COSM10704	Tier-III	No/No
**31**	M/71	KRAS PIK3CA	NM_004985.4 NM_006218.3	c.183A > T p.Gln61His c.1624G > A p.Glu542Lys	Stable	COSM555 COSM125369	Tier-IA Tier-IIC	/Yes
**32**	F/83	KRAS GNAS AKT1 TP53	NM_004985.4 NM_000516.7 NM_005163.2 NM_000546.6	c.35G > A p.Gly12Asp c.602G > A p.Arg201His c.49G > A p.Glu17Lys c.706T > G p.Tyr236Asp	Stable	COSM521 COSM27895 COSM33765 COSM43602	Tier-IA Tier-IB Tier-IB Tier-IB	/Yes
**33**	F/70	No	No	No	Stable	No	No	No/No
**34**	F/58	TP53	NM_000546.6	c.818G > A p.Arg273His	Stable	COSM10660	Tier-III	No/No
**35**	F/63	No	No	No	Stable	No	No	No/No
**36**	F/58	No	No	No	Stable	No	No	No/No
**37**	M/72	KRAS	NM_004985.5	c.38G > A p.Gly13Asp	Stable	COSM532	Tier-IA	/Yes
**38**	M/64	TP53	NM_000546.5	c.438_454del p.Trp146CysfsTer29	Stable	No	TierIII	No/No
**39**	F/67	TP53	NM_000546.5	c.818G > A p.Arg273His	Stable	COSM10660	TierIII	No/No
**40**	M/57	No	No	No	Stable	No	No	No/No
**41**	F/36	KRAS	NM_004985.4	c.436G > A p.Ala146Thr	Stable	COSM19404	TierIA	/Yes
**42**	M/53	TP53	NM_000546.5	c.993 + 2T > A p.(?)	Stable	COSM707067	TierIII	No/No
**43**	M/57	No	No	No	Stable	No	No	No/No
**44**	F/44	BRAF TP53	NM_004333.4 NM_000546.5	c.1799T > A p.Val600Glu c.417G > C p.Lys139Asn	Stable	COSM476 COSM44101	TierIA TierIII	Yes/
**45**	M/42	NRAS TP53	NM_002524.4 NM_000546.5	c.182A > T p.Gln61Leu c.524G > A p. Arg175His	Stable	COSM583 COSM10648	TierIA TierIII	/Yes
**46**	M/42	TP53	NM_000546.5	c.301A > T p.(Lys101*)	Stable	COSM45259	TierIII	No/No
**47**	F/39	KRAS TP53	NM_004985.4 NM_000546.5	c.35G > T p.Gly12Val c.532C > G p.His178Asp	Stable	COSM520 COSM44901	TierIA No	/Yes
**48**	M/61	PIK3CA	NM_006218.3	c.3140A > G p.His1047Arg	Stable	COSM775	TierIIC	No/No
**49**	F/69	No	No	No	Stable	No	No	No/No
**50**	M/69	KRAS	NM_004985.4	c.38G > A p.Gly13Asp	Unstable	COSM532	TierIA	/Yes
**51**	M/55	KRAS TP53	NM_004985.4 NM_000546.5	c.38G > A p.Gly13Asp c.97-8_97-1dup p.Ser33Ilefs* 14	Stable	COSM532 No	TierIA TierIII	/Yes
**52**	M/78	No	No	No	Stable	No	No	No/No
**53**	F/59	No	No	No	Stable	No	No	No/No
**54**	F/58	BRAF TP53	NM_004333.4 NM_000546.5	c.1799T > A p.Val600Glu c.660del p.Tyr220Ter	Stable	COSM476 COSM45582	TierIA TierIII	Yes/
**55**	F/46	No	No	No	Stable	No	No	No/No
**56**	F/62	KRAS	NM_004985.4	c.35G > A p.Gly12Asp	Stable	COSM521	Tier IA	/Yes
**57**	F/53	TP53	NM_000546.6	c.524G > A p.Arg175His	Stable	COSM10648	Tier-III	No/No
**58**	F/79	TP53	NM_000546.6	c.375G > A p.Thr125=	Stable	COSM43904	Tier-III	No/No
**59**	M/73	TP53	NM_000546.6	c.580C > T p.Leu194Phe	Stable	COSM10995	Tier-III	No/No
**60**	M/81	KRAS TP53	NM_004985.5 NM_000546.6	c.38G > A p.Gly13Asp c.527G > A p.Cys176Tyr	Stable	COSM532 COSM10687	TierIA TierIB	/Yes
**61**	M/63	KRAS TP53	NM_004985.4 NM_000546.6	c.35G > A p.Gly12Asp c.560-1G > A p.(?)	Stable	COSM521 COSM43753	Tier-IA Tier-IB	/Yes
**62**	F/71	No	No	No	Stable	No	No	No/No
**63**	M/79	KRAS TP53	NM_004985.5 NM_000546.5	c.35G > T p.Gly12Val c.45_48del p.Ser15ArgfsTer28	Stable	COSM520 COSM5622808	Tier-IA Tier-IB	No/Yes
**64**	M/50	No	No	No	Stable	No	No	No/No
**65**	F/58	KRAS	NM_004985.4	c.38G > A p.Gly13Asp	Stable	COSM532	TierIA	/Yes
**66**	F/45	KRAS NRAS	NM_004985.4 NM_002524.4	c.436G > A p.Ala146Thr c.38G > A p.Gly13Asp	Stable	COSM19404 COSM573	TierIA TierIA	/Yes
**67**	F/61	No	No	No	Stable	No	No	No/No
**68**	M/69	KRAS TP53	NM_004985.5 NM_000546.6	c.183A > C p.Gln61His c.1146del p.Lys382AsnfsTer40	Stable	COSM554 COSM13747	Tier-IA Tier-IB	/Yes
**69**	M/67	KRAS TP53	NM_004985.5 NM_000546.6	c.35G > T p.Gly12Val c.524G > A p.Arg175His	Stable	COSM520 COSM10648	Tier-IA Tier-IB	/Yes
**70**	M/44	TP53 ERBB2 (CNV Fold Change)	NM_000546.6	c.673-2del	Stable	COSM10460500	Tier-III	
**71**	F/56	AKT1 TP53 TP53	NM_005163.2 NM_000546.6 NM_000546.6	c.49G > A p.Glu17Lys c.817C > T p.Arg273Cys c.267del p.Ser90ProfsTer33	Unstable	COSM33765 COSM10659 COSM18610	Tier-IIC Tier-III Tier-III	
**72**	F/74	KRAS	NM_004985.5	c.27_29dup p.Gly10dup	Stable	No	Tier-IID	
**73**	M/44	TP53	NM_000546.6	c.637C > T p.Arg213Ter	Stable	COSM10654	Tier-III	

## Data Availability

The raw sequencing data contains potentially identifiable patient information and therefore cannot be publicly shared in accordance with institutional ethics approval and data protection regulations. Aggregated and anonymized variant data are provided within the manuscript.
